# Limitations of using surrogates for behaviour classification of accelerometer data: refining methods using random forest models in Caprids

**DOI:** 10.1186/s40462-021-00265-7

**Published:** 2021-06-07

**Authors:** Eleanor R. Dickinson, Joshua P. Twining, Rory Wilson, Philip A. Stephens, Jennie Westander, Nikki Marks, David M. Scantlebury

**Affiliations:** 1 School of Biological Sciences, Institute for Global Food Security, Queen’s University Belfast, 19 Chlorine Gardens, Belfast, BT9 5DL Northern Ireland, UK; 2 Biosciences, College of Science, Swansea University, Singleton Park, Swansea, SA2 8PP Wales, UK; 3 Conservation Ecology Group, Department of Biosciences, Durham University, South Road, Durham, DH1 3LE UK; 4 Kolmården Wildlife Park, SE-618 92 Kolmården, Sweden; 5 Öknaskolans Naturbruksgymnasium, SE-611 99 Tystberga, Sweden

**Keywords:** Tri-axial accelerometry, Tri-axial magnetometry, Behaviour identification, Biologging, Alpine ibex, Pygmy goat, Terrain slope

## Abstract

**Background:**

Animal-attached devices can be used on cryptic species to measure their movement and behaviour, enabling unprecedented insights into fundamental aspects of animal ecology and behaviour. However, direct observations of subjects are often still necessary to translate biologging data accurately into meaningful behaviours. As many elusive species cannot easily be observed in the wild, captive or domestic surrogates are typically used to calibrate data from devices. However, the utility of this approach remains equivocal.

**Methods:**

Here, we assess the validity of using captive conspecifics, and phylogenetically-similar domesticated counterparts (surrogate species) for calibrating behaviour classification. Tri-axial accelerometers and tri-axial magnetometers were used with behavioural observations to build random forest models to predict the behaviours. We applied these methods using captive Alpine ibex (*Capra ibex*) and a domestic counterpart, pygmy goats (*Capra aegagrus hircus*), to predict the behaviour including terrain slope for locomotion behaviours of captive Alpine ibex.

**Results:**

Behavioural classification of captive Alpine ibex and domestic pygmy goats was highly accurate (> 98%). Model performance was reduced when using data split per individual, i.e., classifying behaviour of individuals not used to train models (mean ± sd = 56.1 ± 11%). Behavioural classifications using domestic counterparts, i.e., pygmy goat observations to predict ibex behaviour, however, were not sufficient to predict all behaviours of a phylogenetically similar species accurately (> 55%).

**Conclusions:**

We demonstrate methods to refine the use of random forest models to classify behaviours of both captive and free-living animal species. We suggest there are two main reasons for reduced accuracy when using a domestic counterpart to predict the behaviour of a wild species in captivity; domestication leading to morphological differences and the terrain of the environment in which the animals were observed. We also identify limitations when behaviour is predicted in individuals that are not used to train models. Our results demonstrate that biologging device calibration needs to be conducted using: (i) with similar conspecifics, and (ii) in an area where they can perform behaviours on terrain that reflects that of species in the wild.

**Supplementary Information:**

The online version contains supplementary material available at 10.1186/s40462-021-00265-7.

## Introduction

Biologging has transformed what we know about wild animal behaviour [1–3], with particular value attributed to tri-axial body acceleration [4–6]. Biologging devices enable researchers to gain detailed insights into the movement and behaviour of animals [7, 8]. Specifically, where data are limited by direct observations [9] or telemetry is constrained (e.g. sampling intervals are low [10], location is inaccurate [11, 12]), these devices record body movement of animals at high frequencies. They can thus provide detailed information on the study subjects, representing a powerful opportunity to study enigmatic species [6].

Accelerometry data are generally collected at high frequencies (typically tens of hertz), generating large datasets. However, the ease with which these data can be collected is in stark contrast to the difficulties in analysing and interpreting such large data sets (e.g. 40 Hz sampling frequency gives nearly 3.5 million data points per day for a single channel) [13, 14]. Various computational approaches can be used to analyse these data for behavioural identification, including machine-learning algorithms such as k-nearest neighbour [15], random forest models [5], gradient-boosting machines [16], support vectors machines and artificial neural networks [4, 17]. Random forest models are a commonly used approach for classification of behaviours from accelerometry data and provide high accuracy [4, 18].

Whilst the high recording frequencies of the devices are key to identifying behaviours accurately, the use of lower recording frequencies can extend deployment time and reduce associated computational time [18, 19]. The optimisation of sampling frequencies, which will vary with study subject and aims, is therefore an important issue. This is amplified for devices recording parameters other than just acceleration, such as tri-axial magnetometry and barometric pressure [1], which may also be important keys to identifying behaviours [20, 21]. Even when using accelerometry alone, a large number of variables can be computed to include in models for behaviour classification (e.g. 25 variables [5]). Thus, it is important to consider the biological and mechanistic relevance of all variables included in behavioural classification.

Despite the potential of computational approaches to help automate behavioural classification, direct visual observation of the study individuals remains important for the development of accurate algorithms [5]. To overcome the difficulties of observing elusive wild animals, it has been suggested that captive conspecifics can be used to identify behaviours [17]. Indeed, this technique has been shown to have value for measuring behaviour in a range of species [5, 22–24], and where captive individuals are not available, domestic counterparts have been suggested as a viable proxy [25]. However, individual variation [26], including differences in morphology and body-size [25] and the effect of variation in free-living animal habitat compared to domestic and captive settings [22, 27], may be critical when applying such methods. Importantly, it is particularly problematic to test the value of domestic surrogates for wild animals if those wild animals cannot be observed for verification. For example, applying the common method for splitting data into training and validation data sets overestimates the accuracy of models when tested on new individuals because the models are validated on individuals also used to train the model [28].

While it is well acknowledged that differential environment use is an important part of the behavioural ecology of free-living animals [29], it is less appreciated that terrain substrate, superstrate (defined as any material an animal must push against to move [30]), and gradient, affect accelerometer signals and, thereby, the ability to derive behaviours from accelerometry data [27]. For example, the gradient of a terrain should be identifiable in tetrapods because the static acceleration, indicating animal orientation, will change accordingly [31] and animals may, in any event, change gait, stride length and speed according to terrain slope [32, 33], all of which can be manifest in a tri-axial accelerometer signal.

The Alpine ibex (*Capra ibex*) is a Caprid that lives at high altitudes in the central European Alps [34] in populations that are highly fragmented due to pressure from land-use change, agriculture, human disturbance and climate change [35]. Climate change is considered to be particularly important since this species is sensitive to heat and avoids heat stress, which reduces the quality of the food resources they can access [36, 37]. Given on-going global warming, there is concern that physiological and behavioural constraints on the Alpine ibex will lead to severe declines of the species following rapid truncation of suitable habitat [37]. Research is needed to understand the species capacity to adapt to changing environmental conditions, and animal-attached logging systems are ideal for this purpose. However, the high-altitude habitat of the ibex makes it implausible to observe the species in the wild to validate accelerometer signals for behaviour, so it is appropriate to consider using captive surrogates for this. Captive populations of the Alpine ibex are few and access is limited, so a pragmatic approach would be to attempt to calibrate behavioural data using a similar but tractable and accessible species such as the domestic pygmy goat (*Capra aegagrus hircus*), which is phylogenetically similar and readily available in domestic settings [38].

In this study, we tested the validity of this approach by using loggers that measure tri-axial acceleration and magnetic compass heading, on both captive pygmy goats and captive Alpine ibex to examine behaviours of both species using a random forest model approach. We hypothesized that observations of pygmy goat behaviours could be used to predict the behaviours of captive Alpine ibex thereby demonstrating that domestic surrogates can serve as suitable proxies for helping resolve behaviour based on acceleration in rare or difficult-to-handle wild species of conservation concern. We additionally provide a widely applicable template for refining the use of random forest models to predict behaviours including; feature selection approaches, the addition of tri-axial magnetometry variables, selecting the optimum sampling frequency, handling unbalanced observations and data splitting method (random vs individual). With these models, we then aimed to provide behavioural templates for both Alpine ibex and pygmy goats, including predicting the terrain slope for locomotion behaviours. Finally, we examine the ability of our models from one species to predict behaviour in the other in order to assess the value of using surrogate species when captive populations of the focal species are not available for study.

## Methods

### Study subjects and enclosure

The study was conducted using collar-attached ‘Daily Diary’ tags (Wildbyte Technologies Ltd., Swansea, UK [1];) deployed on African pygmy goats at Belfast Zoo (Northern Ireland, UK) in November 2017 and May 2018, and captive Alpine ibex at Kolmården Wildlife Park (Norrköping, Sweden) in November 2018 and November 2019 (Additional file 1 Table S1). At Belfast Zoo, ‘Daily Diary’ tags were deployed on nine female pygmy goats (mean body weight = 25.9 kg, age range = 3–10 years) for periods of 5 days over 1 month within each of two enclosures. Keepers were able to handle the goats to deploy collars. The first enclosure consisted of a sloping grass paddock (slope gradient = 18%, area = 2210 m^2^ [50.1 × 35.3 m]) surrounded by hedges, and the second enclosure was a flat smaller concrete yard with an area of wood mulch (area = 163 m^2^ [16.6 × 7.3 m]).

At Kolmården Wildlife Park, in November 2018, collar-attached devices were deployed on two male Alpine ibex (weight not known, age = 9 years) following a protocol in which the animals were trained though positive reinforcement (using feed pellets as a reward) to wear collars without the need for anaesthesia. Stations to protect the zoo personnel were constructed from wood and both individuals were trained incrementally, over a period of 2 months (Additional file 1 Table S2, *pers comm* Pieter Giljam, Zoospenseful and Kolmården Wildlife Park). Collars were deployed on male Alpine ibex for two periods of 5 days over a month.

In November 2019, collar-attached devices were also deployed on four female Alpine ibex (mean body weight = 45.6 kg, age range = 5–13 years) for a period of 15 days. Female ibex were not compliant to training. Therefore, each individual was sedated using an intramuscular injection of butorphanol (0.009 mg/kg), Etorphine (0.009 mg/kg) and Xylazine (0.674 mg/kg). The collar was deployed, and subject body mass, limb length and horn length recorded. To reverse the anaesthesia, individuals were given an intramuscular injection of naltrexone (0.674 mg/kg) and atipamezole (0.112 mg/kg). Sedation was repeated at the end of the data collection period (after 15 days) to remove the collars. Procedures were conducted by the Kolmården veterinarians. The enclosure was a large area (18,342 m^2^ [202.4 × 80.4 m]) consisting of a mixture of grass and rock surfaces with multiple slopes (range of slopes = 1.7–87%).

### Acceleration data

**Fig. 1:**
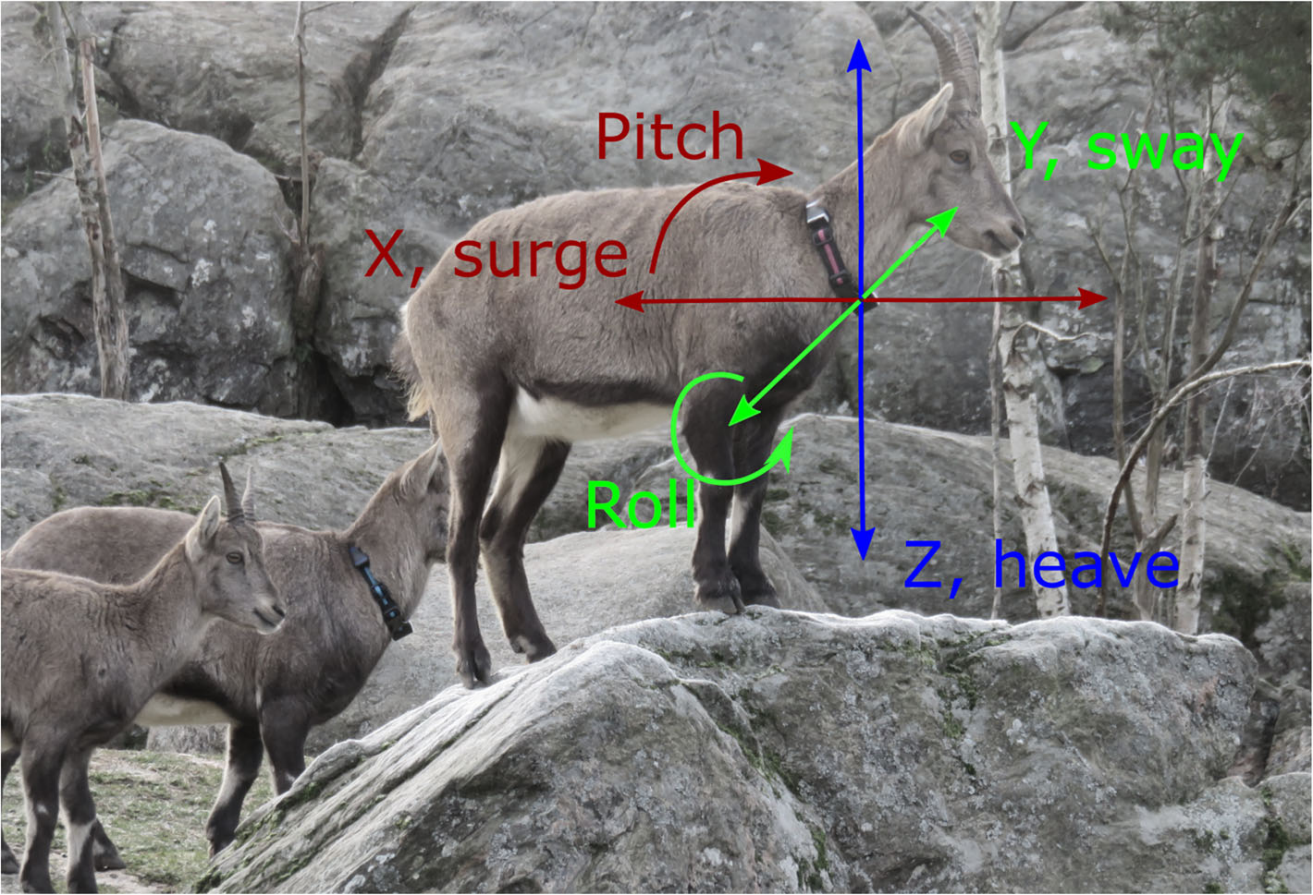
Captive Alpine ibex with a collar-attached ‘Daily Diary’ tag, with a tri-axial accelerometer and magnetometer, depicting the three orthogonal axes (X, Y, Z) recorded at 40 Hz. Pitch and Roll, which are derived from the static acceleration of the X and Y axes [1], respectively, are shown (Photo: Dickinson, E.R.)

Tri-axial acceleration was recorded at a frequency of 40 Hz as well as tri-axial magnetometry, temperature, pressure, time and date. Devices were encased in a plastic housing with a 3.6 V battery (LS 14250, Saft, France; 147 mm × 25 mm; 9 g) and sealed with tesa tape (Tesa® tape 4651, Tesa, Germany). Devices were then attached to the collar using tesa tape and collars were weighted either side of the device to ensure it remained in position on the ventral side of the animal (weight = 135–235 g; dependant on the collar size). Collar weight was within 0.8% of individual body weight and collars were fitted to have a circumference that was 5 cm larger than that of the neck [39]. All devices were oriented so the z-axis corresponded to ‘heave’ (up-down motion), x-axis to ‘surge’ (forward-back motion) and y-axis to ‘sway’ (left-right motion) (Fig. 1). Before deployment, each device was calibrated to the exact time, orientation of the axes and to correct accelerometer and magnetometer offsets.

### Observation and processing of data

To classify behaviour, observations were conducted using a video camera (Canon PowerShot SX720 HS; Canon Inc., Japan). Nine behaviours were distinguished for each species (Table 1) and were recorded for an average of 125.9 min (range: Pygmy goats = 1–221.6 min, Alpine ibex = 2.7–145.2 min). The slope of terrain for locomotion behaviour was also recorded as flat (− 2.5° to 2.5°), uphill (> 2.5°) or downhill (< − 2.5°: Table 1). Individuals were observed from outside their enclosure. Pygmy goats were recorded for a total of 654 min (mean ± sd = 73.5 ± 25.3 min per individual) and Alpine ibex were observed for a total of 516 min (mean ± sd = 87.0 ± 14.4 min per individual) (see Additional file 1 Table S3). Acceleration data were manually labelled according to the observed behaviour for the duration of the observation period using ‘Daily Diary Multiple Trace’ software (Wildbyte Technologies Ltd., Swansea, UK). Only data with labelled behaviour observations were included in the analysis.

**Table 1:** Ethogram of recorded behaviours, including descriptions, for both Alpine ibex and pygmy goats, including the total time, mean time and standard deviation (SD) in seconds observed for each species. Locomotion behaviours were subdivided depending on the slope of terrain. Alpine ibex were not recorded browsing as all their food available was on the floor e.g. grass, hay or pellets. Pygmy goats were not observed climbing due to the lack of a climbing aspect in their enclosures

Behaviour	Description	Alpine ibex			Pygmy goat		
		Total time (s)	Mean time (s)	SD (s)	Total time (s)	Mean time (s)	SD (s)
**Standing**	Stationary in an upright position	8714.1	1452.4	788.9	8665.3	962.8	315.9
**Resting**	Stationary in a laying down position	6165.9	1027.6	648.9	7863.6	982.9	1015.3
**Eating**	Grazing or consuming food from the floor	8104.7	1350.8	640.7	13,295.9	1477.3	756.4
**Browsing**	Consuming food and reaching on hind legs	–	–	–	1953.5	217.2	412.3
**Aggression**	Aggression to or from another individual	590.7	98.5	91.1	296.9	33.0	19.9
**Grooming**	Scratching own body or against another object	242.7	40.4	43.4	428.5	53.6	61.9
**Shaking**	Moving body vigorously to shake	164.0	27.3	16.4	57.8	6.4	5.3
**Walking (Flat, Uphill, Downhill)**	Locomotion in a slow four beat gait	6027.7 (4704.2, 668.4, 655.2)	1004.6 (784.0, 111.4, 109.2)	118.3 (126.9, 49.5, 48.9)	5952.8 (4544.3, 649.0, 759.6)	661.4 (504.9, 81.1, 94.9)	216.1 (158.5, 41.2, 57.0)
**Trotting (Flat, Uphill, Downhill)**	Locomotion in a two beat gait	327.1 (264.9, 20.7, 41.4)	54.5 (44.2, 6.9, 13.8)	41.5 (28.9, 8.0, 10.6)	530.1 (433.9, 28.2, 68.1)	58.9 (54.2, 7.0, 13.6)	45.0 (32.2, 3.8, 15.5)
**Running (Flat, Uphill, Downhill)**	Locomotion in a canter or gallop gait	332.9 (259.2, 34.0, 39.7)	55.5 (43.2, 6.8, 13.2)	41.1 (38.1, 38.1, 10.7)	254.8 (240.9, 9.2, 4.7)	28.3 (26.8, 3.1, 2.4)	16.8 (14.8, 1.5, 1.5)
**Climbing (Uphill, Downhill)**	Travelling on a steep slope with obstacles and steps including jumping up or down steps.	338.4 (160.0, 178.4)	28.2 (26.7, 29.7)	22.55 (21.0, 24.1)	–	–	–

### Accelerometry and magnetometry variables

To classify specific behaviours, 39 variables that are commonly used to detect behaviours from data [1, 5, 21, 26] were extracted or derived from the raw tri-axial acceleration and magnetometry data (Additional file 2 Table S3). From tri-axial acceleration, these variables were either based on static acceleration (cf. Shepard et al. [40]), which describes the orientation of the device relative to gravity and thus the posture of the animal, or dynamic acceleration, which describes the body movement of the animal [41]. From the tri-axial magnetometry, five variables were included, calculated using each of the three orthogonal axes independently or by combining all three axes to provide a measurement of full body motion [20, 21] (Additional file 2 Table S3).

### Building random forest models

Random forest models, which are an extension of classification (decision) trees and are robust and powerful for this type of analysis [42], were built to predict behaviour for both the pygmy goat and Alpine ibex data separately, using accelerometry and magnetometry variables (see above). All analyses were conducted in R version 3.9 [43] using the package *randomForest* [44]. Random forest models use classification trees to classify the observations into different behaviours by building a hierarchy of decision rules based on the variables selected [5, 42]. Our random forest model used 500 iterations (the number of classification trees sampled), and a random subset of data was used to build each tree (bootstrapping) to enable a robust model which limits overfitting and problems associated with unbalanced datasets, which may be common in observations of animals that are likely to spend more time resting than active [5, 26], although unbalanced observations may lead to bias towards dominant observations classes [22]. If an observation is randomly selected, the Gini index measured the probability of it being classified incorrectly. At each classification node, observations were continuously subdivided until the Gini index did not decrease [5, 26]. The mean Gini decrease gave the importance of each variable in classifying the behaviours, with higher values indicating higher importance. The proportionate error of each model (number of misclassifications/number of observations according to the number of trees) was checked for each behaviour and the ‘out-of-bag’ error estimates (observations not included in the bootstrapped sample or tree) examined for each model to evaluate model performance (Additional file 2 Fig. S4).

Models were built with data subsampled at different sampling frequencies to check the effect on classification accuracy of behaviours; 40, 20, 10, 5 and 1 Hz [24]. Random forest models need variables that are not correlated and contribute to the power of the model [45, 46]. To remove correlated features, accelerometry and magnetometry variables were tested for correlation using the Caret package [47]. Correlated variables (Pearson’s r ≥ 0.70) that were the least important according the mean Gini decrease were excluded. Although a consensus does not yet exist on the best methods for random forest model simplification or variable reduction in ecology [48], we removed redundant features using recursive feature elimination (RFE) which fits the random forest models using cross-validation and selects the features to be retained in the model. Variable reduction was conducted consistently for both species models to ensure models used the same variables. The importance of including magnetometry variables was tested separately by removing them from the model and comparing the output for each model using model performance metrics. A general linear model was used to test the effect of sampling frequency and magnetometry variable inclusion on classification accuracy. Model accuracy was included as the response variable and sampling frequency, species and data (accelerometry or accelerometry and magnetometry) included as explanatory variables.

The following steps were conducted with data at the lowest sampling frequency that resulted in a high classification accuracy, bearing in mind that unbalanced datasets may bias the predictive ability of classification methods toward the most dominant data classes [22] and that standing, eating, browsing, walking and resting had a higher number of observations than other behaviours (see Table 1). We used a down-sampling strategy to handle imbalanced data classes for relevant behaviours to remove instances in the majority classes. Specifically, behaviour classes that were observed for longer than the median (560.4 s) were down-sampled randomly using the Caret package [47]. Another strategy that may improve model performance is reducing the number of behaviour categories. The initial models included all behaviours observed in each species, and the effect of reducing the number of behaviours was tested by removing those assumed to be less relevant to ethological studies: aggression, grooming, and shaking.

Authors using random forest models to predict behaviour from accelerometry generally split data randomly into 60% training and 40% validation sets (e.g. [5, 26]). However, the value of using data split per individual datasets has been highlighted when validating the ability of models to predict behaviour of unobserved individuals [28]. In this study, we built two model sets, the first splitting the data 60/40 randomly, with data from each individual present in both the training and the validation models, and the other approximately split 60/40 at the individual level, with individuals only in either the training or validation sets. The individual-split models were repeated for all combinations of individuals in the training or validation data sets using a k-fold cross-validation strategy to give average model performance [28] (Table 1). The effect of balancing observations, and reduced number of behaviour classes on the model performance metrics was tested for both the random and individual-split models using one-way ANOVAS and Tukey pairwise-comparisons for each species.

### Random forest model validation

To estimate model performance for each random forest model used in this study, confusion matrices were produced for the model on the validation dataset, highlighting true positives, false positives and false negatives [5, 27]. From these, the model accuracy, precision and recall were calculated using the number of true positives (TP, correctly classified positive behaviours), false positives (FP, incorrectly classified positive behaviours), true negatives (TN, correctly classified negative behaviours), false negatives (FN, incorrectly classified negative behaviours). Model accuracy was calculated as the percentage of true positives and true negatives [28]:

1 $$ Accuracy=\frac{TP}{TP+ FP+ TN+ FN} $$

Precision was defined as the proportion of positive classifications that were true compared to false positives:

2 $$ Precision=\frac{TP}{TP+ FP} $$

Recall was defined as the proportion of positive classifications that were true compared to the false negatives [15]:

3 $$ \mathrm{R} ecall=\frac{TP}{TP+ FN} $$

The F1 statistic was then calculated as the harmonic mean of Precision and Recall used as a metric of the overall performance for classification of each behaviour [26]:

4 $$ F1=\frac{2}{\frac{1}{Precision}+\frac{1}{Recall}} $$

### Predicting across species

To determine whether pygmy goats could be used as a surrogate species to predict Alpine ibex behaviour, the model using the pygmy goat dataset was used to predict Alpine ibex behaviour from the Alpine ibex dataset. Behaviours that were not observed across both species (specifically, climbing and browsing) were excluded. Models with data at the lowest acceptable sampling frequency were used to predict behaviour and, for locomotory behaviours, behaviour subdivided by slope of terrain (flat, uphill or downhill; see Table 1). Model performance was compared with the full initial model to when data observations classes were balanced and the number of predicted behaviours was reduced. A sex-specific model was tested that excluded the male ibex from the cross-species model. To check model performance compared to a random model, observed behaviours were randomly generated onto the acceleration data using the same proportion of actual observations for each behaviour and used to build a random forest model.

## Results

### Refining random forest models

**Fig. 2:**
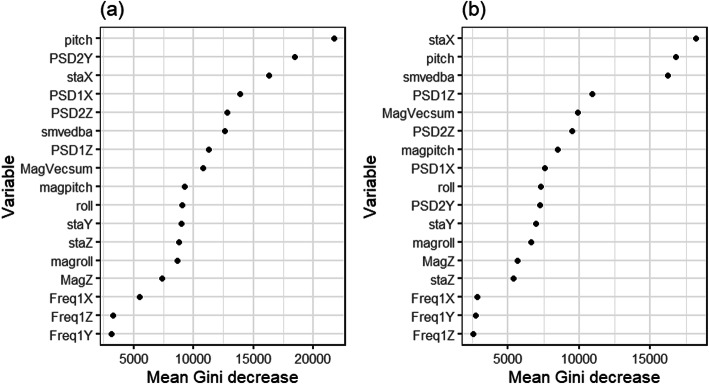
The mean Gini decrease of the variables used to predict behaviour, ordered by importance to the model: (**A**) Alpine ibex and (**B**) pygmy goat, with the reduced variables included in the final model

**Fig. 3:**
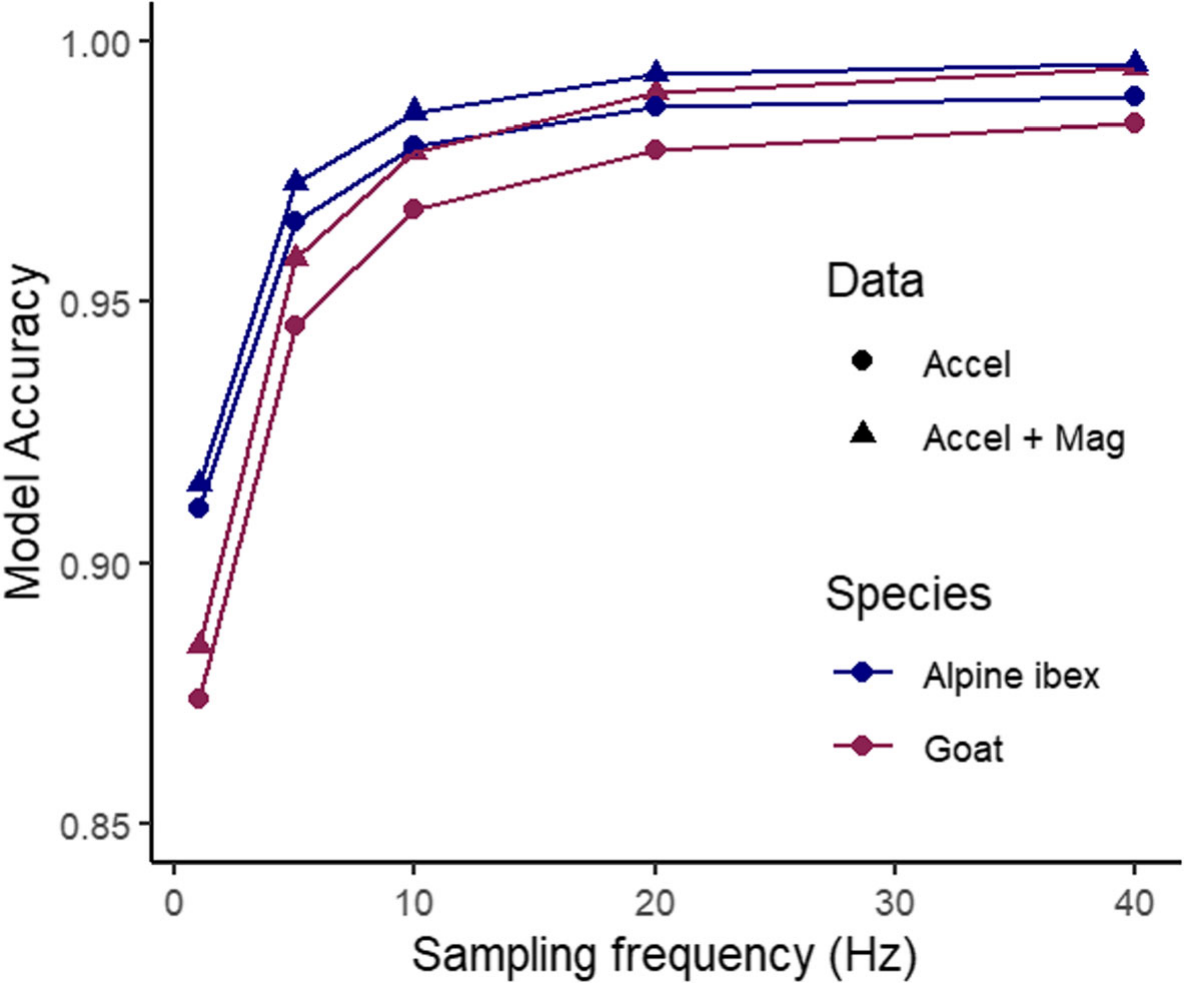
Model accuracy of Random Forest models to predict the behaviour of Alpine ibex and pygmy goats, using either accelerometry variables or accelerometry and magnetometry variables at different sampling frequencies (1, 5, 10, 20 and 40 Hz)

Random forest models were built for the different sampling frequencies using either accelerometry variables only or both accelerometry and magnetometry variables. Seven variables were removed due to them being highly correlated and a further 13 variables were removed in RFE, with 17 variables included in the final model (Fig. 2; Additional file 2 Fig. S4). Model accuracy was not significantly different between the 40 Hz and the 20 Hz model (t_4,5_ = − 0.003, *p* = 0.71) or the 10 Hz model (t_4,5_ = − 0.013, *p* = 0.21). However, it was significantly lower at 5 Hz (t_4,5_ = − 0.030, *p* = 0.025), and 1 Hz (t_4,5_ = − 0.095, *p* < 0.001) (Fig. 3). Thus, a sampling frequency of 10 Hz was selected as the best model as a compromise between model performance and ability to process. Overall, model accuracy was significantly different for Alpine ibex and pygmy goats (t_6,13_ = − 0.13, *p* = 0.001).

**Fig. 4:**
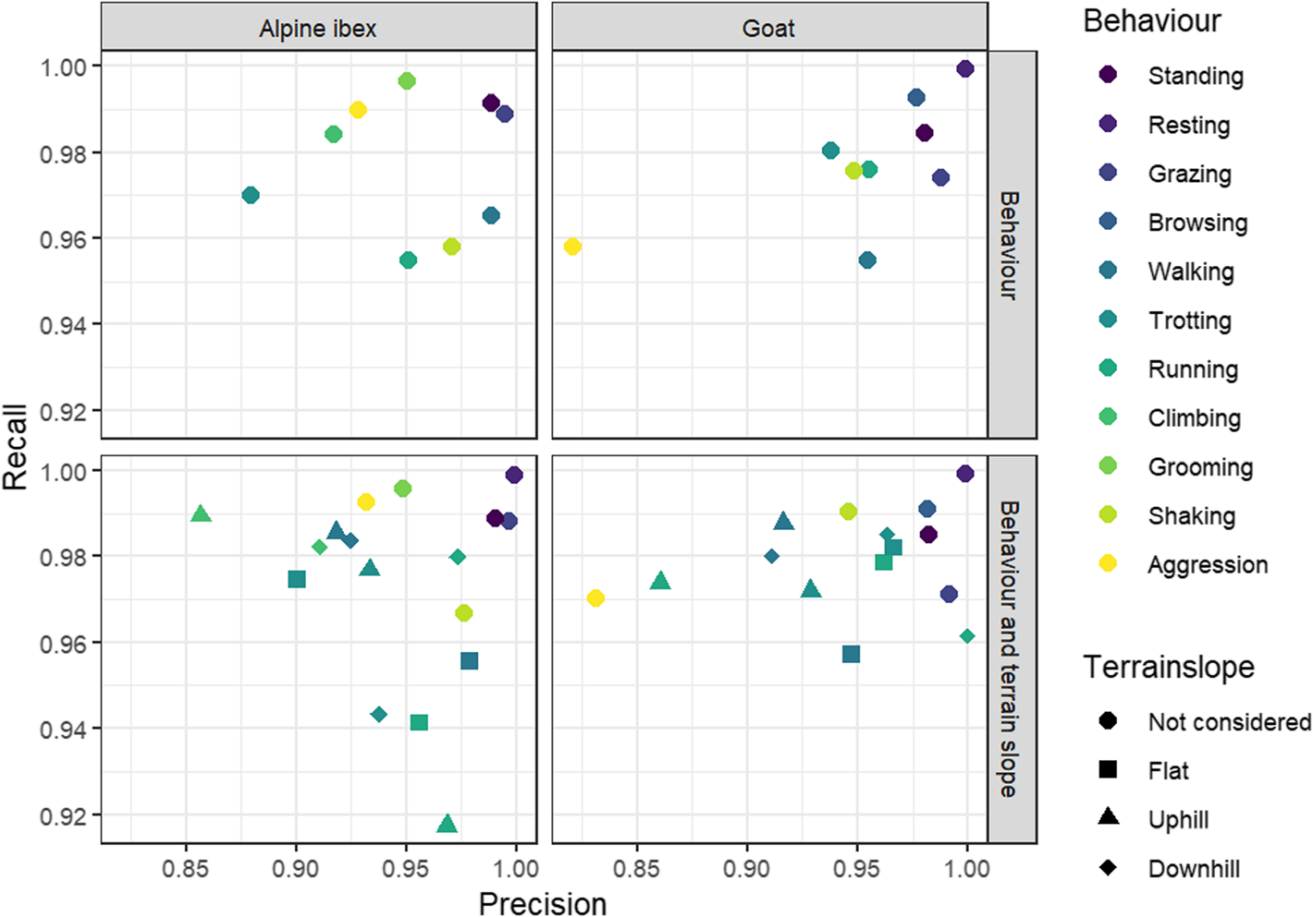
Precision and Recall of each behaviour categorised in the models for Alpine ibex and pygmy goats. Terrain slope is predicted for various locomotion behaviours in the bottom panel

Comparing models with a sampling frequency of 10 Hz and higher, model accuracy was higher when magnetometry variables were included (t_2,9_ = 0.008, *p* = 0.03). Model accuracy of the final selected models using randomly split data was 98.6% for Alpine ibex with a mean ± SD F1 statistic of 0.96 ± 0.011 and 97.8% for pygmy goats with a mean ± SD F1 statistic of 0.96 ± 0.016 (Table 2). Although model accuracy was lower using balanced data classes (F_1,2_ = 0.079, *p* = 0.80), the precision for separate behaviours was significantly higher (F_1,2_ = 72.9, *p* = 0.013). Prediction of behaviours using fewer behaviours enhanced model accuracy (F_1,2_ = 0.17, *p* = 0.72) and the mean F1 statistic (F_1,2_ = 12.45, *p* = 0.07). Using data split per individual, the mean model accuracy was 56.7 ± 0.06% for Alpine ibex with a mean ± SD F1 statistic of 0.37 ± 0.02 and 57.9 ± 0.05% for pygmy goats with a mean ± SD F1 statistic of 0.34 ± 0.03 (Table 2; Fig. 4). Model accuracy was significantly lower in balanced data classes (F_1,28_ = 46.6, *p* < 0.001) and was improved when the number of behaviour classes was reduced (F_1,28_ = 0.70, *p* = 0.41). Using F1 statistic as a measure of model performance, model performance was higher when using balanced observations (F_1,28_ = 3.71, *p* = 0.06) and when the number of behaviours was reduced (F_1,28_ = 25.3, p < 0.001).

**Table 2:** The overall model accuracy and mean F1 statistic (harmonic mean of the precision and recall) for each 10 Hz model using different strategies to build the random forest model. *SD not available

Model	Pygmy goat		Alpine ibex	
	Accuracy ± SD (%)	F1 statistic ± SD	Accuracy ± SD (%)	F1 statistic ± SD
Random split train and test data	97.8	0.96 ± 0.02	98.6	0.96 ± 0.01
with balanced observations	97.6	0.98 ± 0.02	98.6	0.99 ± 0.01
with reduced behaviours	98.2	0.97 ± 0.02	98.7	0.97 ± 0.01
Data split per individual train and test data	57.8 ± 5.4	0.34 ± 0.03	65.5 ± 5.2	0.40 ± 0.02
with balanced observations	42.1 ± 6.0	0.38 ± 0.05	47.7 ± 7.2	0.43 ± 0.05
with reduced behaviours	59.2 ± 5.7	0.43 ± 0.05	68.6 ± 5.1	0.51 ± 0.02

### Behavioural templates for Alpine ibex and pygmy goats

Random forest models, at a sampling frequency of 10 Hz, were built to predict the slope of the terrain for locomotion behaviours; flat, uphill or downhill. Overall model accuracy when slope was included was 98.6% for Alpine ibex with a mean ± SD F1 statistic of 0.96 ± 0.016 and 98.0% for pygmy goats with a mean ± SD F1 statistic of 0.96 ± 0.016 (Fig. 4; Table 2; Additional file 3 Fig. S6). Pitch was the most important variable for pygmy goats, and smoothed VeDBA was the most important variable for Alpine ibex predicting behaviours. Static X axis acceleration was the most important variable when the model predicted Alpine ibex behaviour including terrain slope.

**Fig. 5:**
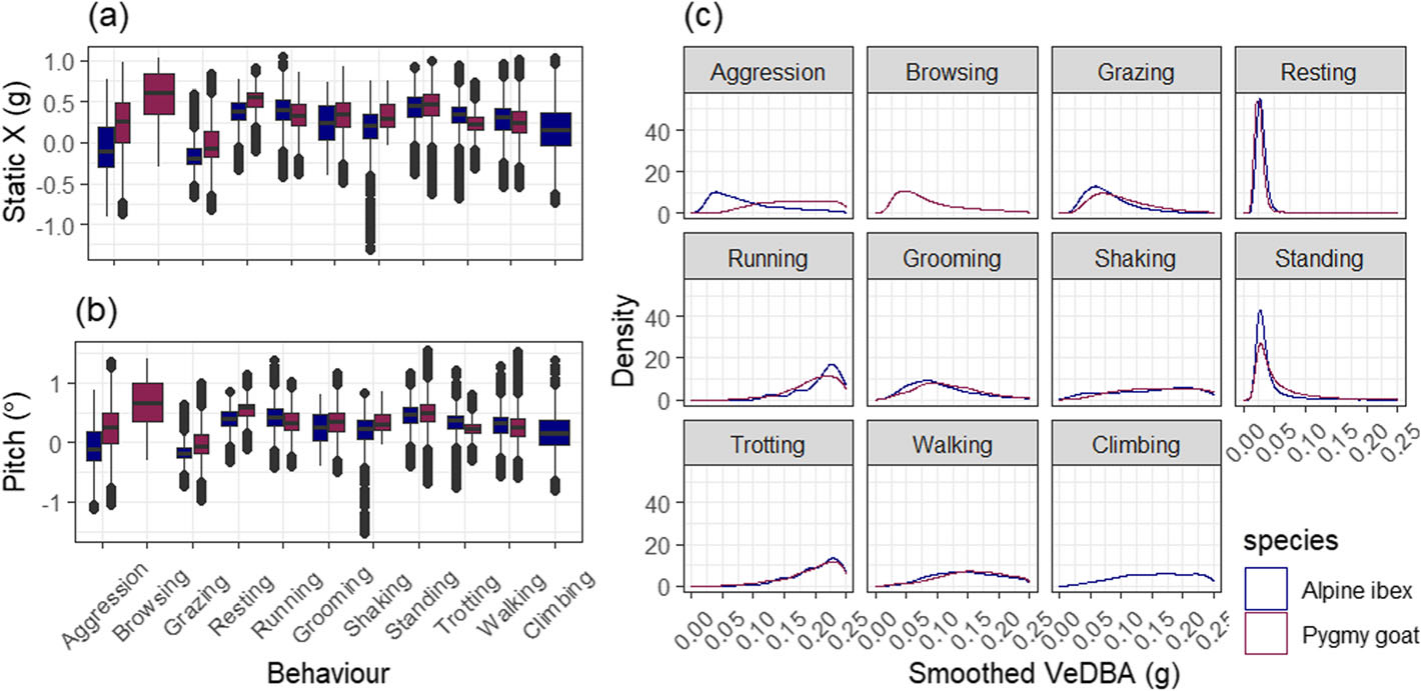
The three variables that were in the top 5 most important variables for differentiating Alpine ibex and pygmy goat behaviour: **A**) static X, **B**) pitch and C) maximum amplitude of oscillations of the sway axis over 2 s (PSD1Y)

Three variables were in the top 5 most important variables, ranked by mean Gini decrease, for both the Alpine ibex and pygmy goats. These were posture, given by the surge axis (static X), angle of surge posture (pitch) and smoothed VeDBA (smVeDBA) (Fig. 5; Additional file 3 Fig. S6 Table S5).

### Applying pygmy goat behavioural template to Alpine ibex

**Fig. 6:**
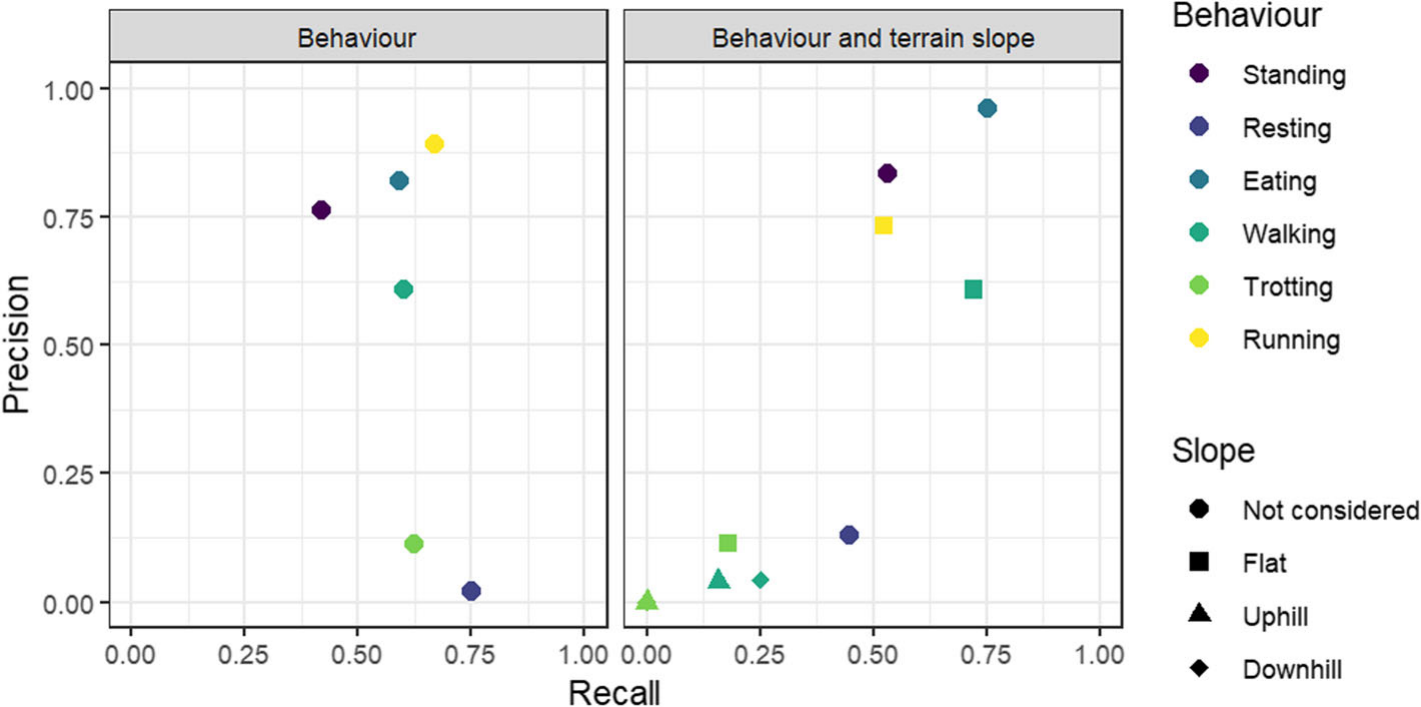
Precision and recall for each behaviour for the model trained with pygmy goat behaviour to predict Alpine ibex behaviour (cf. Figure 4, noting scale differences)

In the investigation examining the extent to which the model conditioned on the pygmy goat training dataset could be used to predict behaviours observed in the Alpine ibex training dataset, model accuracy was 54.3% for predicting behaviours. The model reached a mean ± SD precision of 0.54 ± 0.38, recall of 0.61 ± 0.11 and F1 statistic of 0.47 ± 0.29 (Table 3). The largest errors in the model were produced from misclassifying resting as standing, and trotting as either walking or running (Additional file 3 Table S6). Standing, walking, eating and running had the highest recall and precision in this model (Fig. 6). A model using randomly generated ‘observed’ behaviours had a classification accuracy of 15.4% (Table 3).

**Table 3:** The mean precision, recall and F1 statistic (± SD) for each random forest model predicting behaviour or behaviour including slope of terrain for Alpine ibex and pygmy goats

Model	Classification accuracy	Mean precision	Mean recall	Mean F1 statistic
Random split behaviour	98.3%	0.95 ± 0.05	0.98 ± 0.018	0.96 ± 0.030
Random split behaviour including slope of terrain	98.2%	0.95 ± 0.042	0.98 ± 0.018	0.96 ± 0.024
Data split per individual behaviour	63.0%	0.48 ± 0.32	0.55 ± 0.27	0.46 ± 0.28
Data split per individual behaviour including slope of terrain	68.1%	0.36 ± 0.028	0.42 ± 0.082	0.034 ± 0.046
Pygmy goat predicting Alpine ibex behaviour	55.5%	0.55 ± 0.39	0.62 ± 0.10	0.48 ± 0.30
Pygmy goat predicting female Alpine ibex behaviour	60.2%	0.55 ± 0.39	0.50 ± 0.26	0.49 ± 0.32
Pygmy goat predicting Alpine ibex behaviour including slope of terrain	59.8%	0.29 ± 0.38	0.30 ± 0.29	0.27 ± 0.32
Pygmy goat predicting female Alpine ibex behaviour including slope of terrain	67.8%	0.28 ± 0.42	0.25 ± 0.34	0.26 ± 0.37
Randomly generated behaviours	15.4%	0.010 ± 0.27	0.058 ± 0.09	0.038 ± 0.08
Randomly generated behaviours with slope of terrain	26.4%	0.068 ± 0.24	0.040 ± 0.11	0.041 ± 0.12

Model accuracy for predicting behaviours and slope of terrain for locomotion behaviour was 60.5%. The model reached a mean ± SD precision of 0.28 ± 0.41, recall of 0.26 ± 0.30 and F1 statistic of 0.24 ± 0.34 (Table 3). Locomotion behaviours on a slope had very low precision and recall (Fig. 6; Additional file 3 Table S7). A model using randomly generated ‘observed’ behaviours including slope for locomotion behaviours had a classification accuracy of 26.4% (Table 3). For both models, model accuracy improved when using a sex-specific model (predicting only female Alpine ibex behaviour), however other model performance metrics did not change.

## Discussion

Accurately identifying animal behaviour is key to the validity of using accelerometers to address important ecological questions in free-ranging animals. However, there remains limited information on best practice, especially when captive or domestic individuals are used to inform workers on the putative behaviour of wild species. In this study, behavioural classification was achieved with high accuracy for both captive Alpine ibex and domestic pygmy goats, using observations of each species respectively and taking steps to refine the application of random forest models. All behaviours and the slope of terrain for locomotion behaviours could be predicted with high accuracy. However, limitations were identified when the models were used to predict the behaviour of individuals not used in model training, whether they were the same species or not. Domestic or captive surrogates may be useful to predict the broad behaviours of a captive wild species but locomotion on terrain with different slope characteristics remains problematic. Thus, while captive surrogates may be useful for classifying behaviour in some free-ranging animals, the selection of appropriate counterparts or surrogates must be carefully considered for accurately classifying behaviours.

Despite decreased model performance when Alpine ibex behaviour was predicted from domestic pygmy goats, the biggest decrease in model performance occurred when individually split data was used instead of randomly split data. This suggests that the limitations of predicting the behaviours of individuals that cannot be observed lies within intraspecific individual differences rather than inter-specific variation [26]. Behaviours such as resting were not well identified, which is typically considered to be an easy behaviour to identify, and a definitive explanation for this remains elusive. Despite this, broad behaviours were identifiable although some behaviours remained problematic in the cross-species model, particularly as regards the effect of terrain slope for locomotion- and resting behaviours.

Domestic surrogates, or even captive surrogates of a different species, have been suggested to have value for informing behavioural classification and the concept is certainly logical [22, 25]. Against this though, we observed low classification accuracy, and were unable to identify the full suite of behaviours observed in the captive counterparts, using our domestic surrogate. Critically, the value of using captive or domestic individuals as surrogates to predict the behaviour of free-living individuals requires that the surrogates and wild animals to move and behave in a similar way. However, the extent to which this is true depends critically on the size and morphology differences between the species dyads. For example, domestication may change bone structure [49], thus leading to changes in gait and movement and body size, which can have a marked effect on stride length and stride frequency [50], and with it the acceleration values recorded by animal-attached devices. Pygmy goats are known for their characteristically short legs (height = 31 and 45 cm [51];) associated with their adaptation to humid environments [52], whereas the longer legs of Alpine ibex facilitate locomotion through their mountainous habitat (female height = 73 to 84 cm, male height = 90 to 101 cm [34]). The high degree of sexual dimorphism in Alpine ibex [34], means that males are more different than females to female pygmy goats. This disparity may explain the reduced accuracy of models using pygmy goat observations to predict Alpine ibex behaviour. Indeed, model performance was higher when pygmy goat observations were used to predict the behaviour of female ibex, indicating that it is the increased difference between male Alpine ibex and female pygmy goats that reduces the ability of the model to predict behaviour between them. This suggests that there is value in using sex specific models when classifying behaviours sexually dimorphic species.

The environment in which the surrogate individuals live must replicate, as far as possible, that of their wild counterparts for them to exhibit the same behavioural profiles. Our captive Alpine ibex were observed to display a wider range of behaviours and terrain slopes because they were kept in a large and varied enclosure with rocks and small cliffs. So, simplistically, climbing in ibex could not be predicted using our pygmy goat surrogate because, although the goats had slopes within their enclosure, none were comparable to the rocks that ibex used. This limitation may be especially important for measuring behaviour of individuals that may access food or water in a manner different to that observed in captivity, a clear case being predators that cannot hunt in captivity [24, 28]. In fact, animal home ranges can cover large areas which display habitat and topographical heterogeneity, which will presumably produce corresponding heterogeneity in accelerometer signals, particularly during movement, so it is important to be able to interpret and account for the gradient, substrate and superstrate of the terrain during locomotion [1]. Using surrogates that are in a varied enclosure that mimics the species natural environment would reduce the issues linked to environment that arise from using captive or domestic surrogates.

Orientation on slopes is expected to alter the static surge acceleration signal as the collar-attached device abuts the animal’s neck, particularly if the animal is facing, or moving, up an appreciable slope. Indeed, the extent to which the device on the collar can swing should prove an important issue in defining behaviours; the more it can swing, the more it will act like a gimble and be less likely to be constrained to a particular angle by abutting the neck. Against this, loose collars may introduce unwanted variability during movement [39]. Terrain will also affect the acceleration profiles measured for different behaviours because animals often respond to terrain by changing gait, stride length and speed [53], so enclosures used for captive calibration of behaviours from logging devices should display the entire range of topographies available to the free-ranging animals of interest.

A perennial issue for biologgers is the trade-off between high resolution data (both in terms of time and bits) and required battery power [19, 54]. Lower frequencies can extend deployment time and reduce battery power, memory on internal storage devices and required processing power. In this study, we found that highest classification accuracy was achieved using a sampling rate of 10 Hz or above and, even when sampling rate was reduced to 1 Hz, it still resulted in 87.4% correctly classified behaviours, which is deemed acceptable by other studies [18, 24, 55].

The ease with which biologger data can be analysed to highlight behaviour using random forests [5] belies a few important considerations. Firstly, there is a tendency to include a large number of variables from tri-axial accelerometers for random forest models even though many have not been tested for the benefit of their inclusion. Although random forest models can handle noisy variables and can be robust to overfitting [48], 20 variables were not included in the dataset, either due to being correlated or deemed redundant using recursive feature selection. This suggests that there is value in selecting variables that are biologically and mechanistically important in describing the behaviours and therefore important to the model. This, in turn, necessitates proper understanding of what the various acceleration metrics mean and how they are changed by both the different behaviours and the environment (topography etc.). Other steps that have been suggested to improve random forest model performance were also taken. Although using balanced observation classes did not significantly improve model performance, steps to reduce the number of behaviours predicted (removing less relevant behaviours) did improve model performance. The behaviours included when classifying behaviours should be carefully selected, as including behaviours that are not relevant for the study may reduce the accuracy of relevant behaviours. Furthermore, when applying behaviour templates to unobserved data, steps to reduce the chance of predicting the wrong behaviour should be taken such as setting a threshold accuracy (see Ferdinandy et al., [28]).

Finally, many biologgers have accelerometers within inertial measurement units (IMUs), which also have tri-axial magnetometers built in although few studies have included tri-axial magnetometry in behavioural classification despite the potential for it to be useful [20, 21]. Our work showed that by including (limited) variables derived from tri-axial magnetometry, classification accuracy was significantly improved. This may prove particularly valuable in the future, since magnetometers may be able to elucidate patterns of movement in a manner different to accelerometers, thus potentially providing important additional information for behavioural classification [17].

## Conclusions

A template for applying methods to identify the behaviours of wild or captive Caprids using captive and domestic counterparts using tri-axial accelerometry and magnetometry is provided, highlighting the need the create standardised methodologies, including data processing steps, especially when selecting variables and using random forest models. High model performance could be achieved for two caprid species using video observations with a relatively low sampling frequency (10 Hz), including predicting the slope of terrain for locomotion behaviours. Tri-axial magnetometry is a useful tool to aid behavioural classification and slope of terrain for locomotion behaviours could be accurately predicted. We demonstrate the importance of using sex-split training datasets in sexually dimorphic species. While we show that model performance is reduced when predicting the behaviours of individuals not included in the training data, it is comparable when predicting for the same or a similar species. The use of an individual-split cross-validation approach better demonstrates the application of these methods to individuals of the same or similar species. For prediction of the behaviours of a different species, all efforts should be made to maximise the similarities between surrogate and study species, including their respective environments.

## Supplementary Information

**Additional file 1: Table S1.** Details of the individuals and training the male Alpine ibex to have collars put on and taken off. **Table S2.** Description of training protocol. **Figure S1*.*** A male ibex being rewarded standing in the protective feeding station (step 3). **Figure S3.** Three target male ibex rewarded in their designated protective stations (step 4, only two were successfully trained beyond this step). **Figure S4*.*** Holding the collar around a male ibex neck while he stands in the protective station, one trainer holds the collar while the second provides the reward (step 7). **Table S3*.*** Total time observed of each behaviour for each individual pygmy goat (G) or Alpine ibex (IB) in seconds.

**Additional file 2 **Methods for building and refining random forest models to predict the behaviour of Alpine ibex and pygmy goats. **Table S4.** A list of the accelerometry and magnetometry variables that are used or calculated for the random forest model. Including the name, and label, the description of the variable and its calculation. **Figure S4.** Recursive feature elimination plots showing the cross-validated model accuracy when a different number of acceleration and magnetometry variables are included in the random forest models for classifying the behaviours of (a) Alpine ibex and (b) pygmy goat. **Figure S5.** Random forest error plots across 500 trees for classifying each of the nine behavioural states (Aggression, Browsing (pygmy goats only), Climbing (Alpine ibex only), Grazing, Grooming, Lying down, Running, Shaking, Standing, Trotting and Walking) and Out-of-bag (OOB) error estimates for each different model at 10 Hz for both species (a,b) including the models with: (c,d) balanced observations and (e,f) reduced behaviour classes. **Figure S6.** Random forest error plots across 500 trees for classifying each of the nine behavioural states including terrain slope for locomotion behaviours (Aggression, Browsing (pygmy goats only), Climbing (Alpine ibex only), Grazing, Grooming, Lying down, Running, Shaking, Standing, Trotting and Walking) and Out-of-bag (OOB) error estimates, for (A) Alpine ibex and (B) pygmy goats*.* **Table S5.** The variable reduction process to reach the final selected model.

**Additional file 3: **Random forest model results. **Figure S6.** The importance of each variable retained in the models predicting behaviour and behaviours including terrain slope. **Table S5.** The median and 1st and 3rd quantile of acceleration, for each behaviour and species, for three variables. **Table S6.** Confusion matrix showing the observed behaviours and predicted behaviours (in seconds) when training the random forest model built using the pygmy goat training dataset. **Table S7.** Confusion matrix showing the observed behaviours and predicted behaviours (in seconds) when using a random forest model built using pygmy goat training dataset and tested on the Alpine ibex training data set. **Table S8.** Confusion matrix showing the observed behaviours and predicted behaviours, including the gradient of terrain for locomotion behaviours, when training the random forest model built using the pygmy goat training dataset. **Table S9.** Confusion matrix showing the observed behaviours and predicted behaviours, including the gradient of terrain for locomotion behaviours, when using a random forest model built using pygmy goat training dataset and tested on the Alpine ibex training data set.

**Additional file 4: Figure S6.** The importance of each variable ordered by mean Gini decrease for the model predicting behaviours including slope of terrain; (a Pygmy goats with ‘Pitch’ as the most important variable and (b) Alpine ibex with ‘Static X’ as the most important variable. **Table S5.** The median and 1st and 3rd quantile of acceleration, for each behaviour and species, for the three variables that are in the top 5 most important variables for predicting behaviour of both pygmy goats and Alpine ibex. **Table S6.** Confusion matrix showing the observed behaviours and predicted behaviours when using a random forest model built using pygmy goat training dataset and tested on the Alpine ibex training data set. Italicised cells are the true positives where the behaviour has been correctly predicted. **Table S7.** Confusion matrix showing the observed behaviours and predicted behaviours, including the gradient of terrain for locomotion behaviours, when using a random forest model built using pygmy goat training dataset and tested on the Alpine ibex training data set. Italicised cells are the true positives where the behaviour has been correctly predicted. (Downhill = D, Flat = F, Uphill = U).

## Data Availability

The datasets for this study and the code used for analysis will be made available online.
