# Supplementary material for: Limitations of using surrogates for behaviour classification of accelerometer data: refining methods using random forest models in Caprids
Source: Mov Ecol. 2021 Jun 7;9:28. doi: 10.1186/s40462-021-00265-7 (PMC8186069; doi:10.1186/s40462-021-00265-7)
Supplement: Supplementary file 1 — Additional file 1: Table S1. Details of the individuals and training the male Alpine ibex to have collars put on and taken off. Table S2. Description of training protocol. Figure S1. A male ibex being rewarded standing in the protective feeding station (step 3). Figure S3. Three target male ibex rewarded in their designated protective stations (step 4, only two were successfully trained beyond this step). Figure S4. Holding the collar around a male ibex neck while he stands in the protective station, one trainer holds the collar while the second provides the reward (step 7). Table S3. Total time observed of each behaviour for each individual pygmy goat (G) or Alpine ibex (IB) in seconds. [file 40462_2021_265_MOESM1_ESM.pdf]

Additional file 1: Details of the individuals and training the male Alpine ibex to have collars put on and taken off.

Table S1: Details of individuals including species, sex, age, weight and neck size of each individual. Female Alpine ibex were weighed using hanging scales (Salter, Kent, UK) during sedation. Male Alpine ibex were not weighed but generally weigh between 67 and 117 kg [1]. Pygmy goats were weighed standing on animal weighing scales (Adam equipment, Milton Keynes, UK). Pygmy goats were all observed in both enclosures.

**Species**	**ID**	**Sex**	**Age (years)**	**Weight (kg)**	**Neck size (cm)**	**Time observed**
Alpine ibex	IB01	Male	9	NA	73	
	IB02	Male	9	NA	64	
	IB03	Female	5	44.8	36	
	IB04	Female	5	47.8	40	
	IB05	Female	4	46.3	37	
	IB06	Female	15	43.5	35	
Pygmy goat	G01	Female	6	31.4	38	
	G02	Female	4	23.4	35	
	G03	Female	6	31.5	40	
	G04	Female	10	28.7	36	
	G05	Female	7	23.8	34	
	G06	Female	3	15.1	32	
	G07	Female	3	17.5	30	
	G08	Female	7	31.2	40	
	G09	Female	5	30.6	34	

1. Parrini F, Cain JW, Krausman PR. Capra ibex (Artiodactyla: Bovidae). Mamm Species. 2009;830:1–12.

Table S2: Training was done using wooden protective stations and a bespoke training protocal twice a day before daily feeding times. Training was done using incremental steps with positive reinforcement (feed pellets) over a period of two months. The protective stations were designed so that trainers could work with the ibex safely and there was a target for the animals to come to, so they were not trained to come close to people without barriers for safety. Stations had a step at the front for the ibex to stand on, this raised the height of their necks and meant they could not use their full head force if aggressive. Wooden triangles were attached so the trainers could put their hands to the ibex without risk from the horns. Each target individual has a designated station and feeding trays were removable so reward could be withdrawn when the wrong ibex was in a station.

Step	Description
1	Recall (call the animals to the trainers) with the main aim to associate us with food so they come to trainers when present.
2	Recall at the protective stations (call the animals to the training area). Individuals rewarded when they approached stations.
3	Stand within the designated station.
4	Stand on the step at the front of the protective station and stay in the protective station when a trainer steps next to the station. The clicking noise of the collar was also introduced.
5	Stand while the collar is place in front of the feeding tray and then behind the feeding tray where the ibex must eat over the collar.
6	Stay still over the feeding tray while collar moves and then touches the neck of the ibex.
7	Stay still while the collar is closed around the neck (initially not clicked shut).
8	Stay still while the collar is clicked shut around the neck and then stay still while the collar is removed to ensure that the collar can be retrieved.

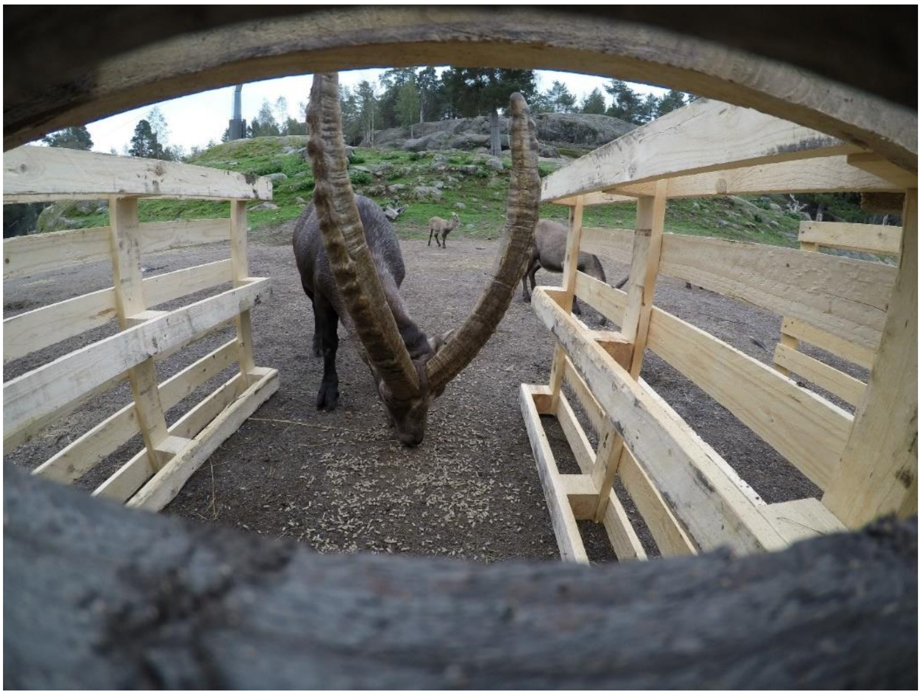

Figure S1: A male ibex being rewarded standing in the protective feeding station (step 3: Photo; Pieter Giljam)

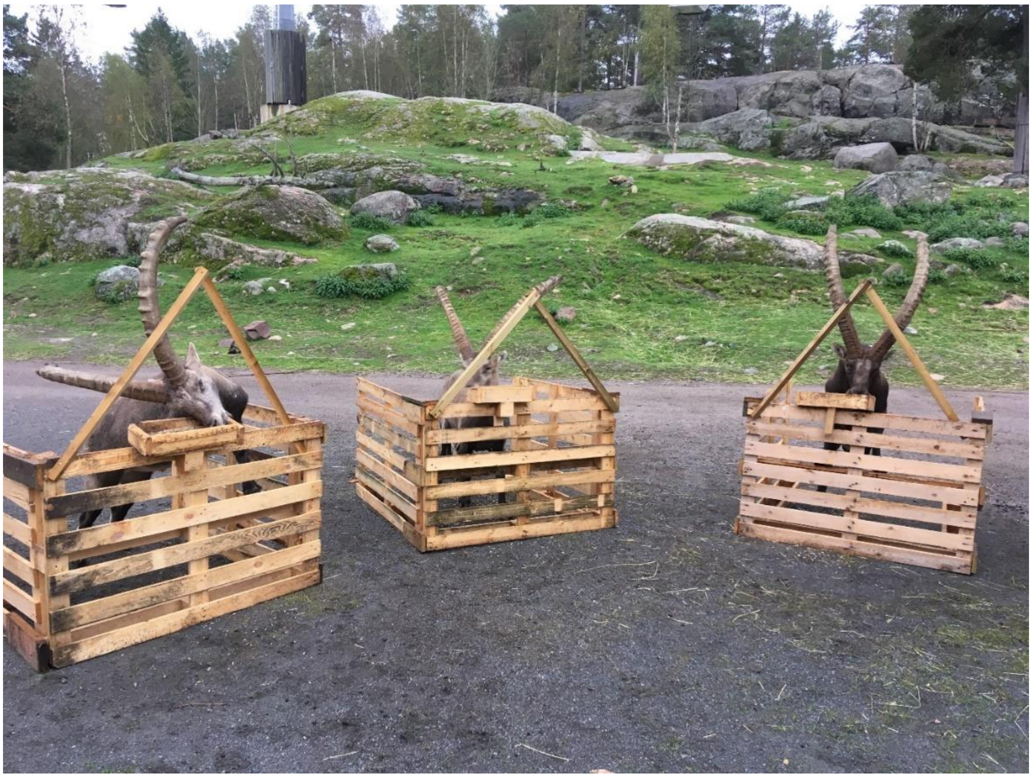

Figure S3: Three target male ibex rewarded in their designated protective stations (step 4, only two were successfully trained beyond this step: Photo; Dickinson, E.R.)

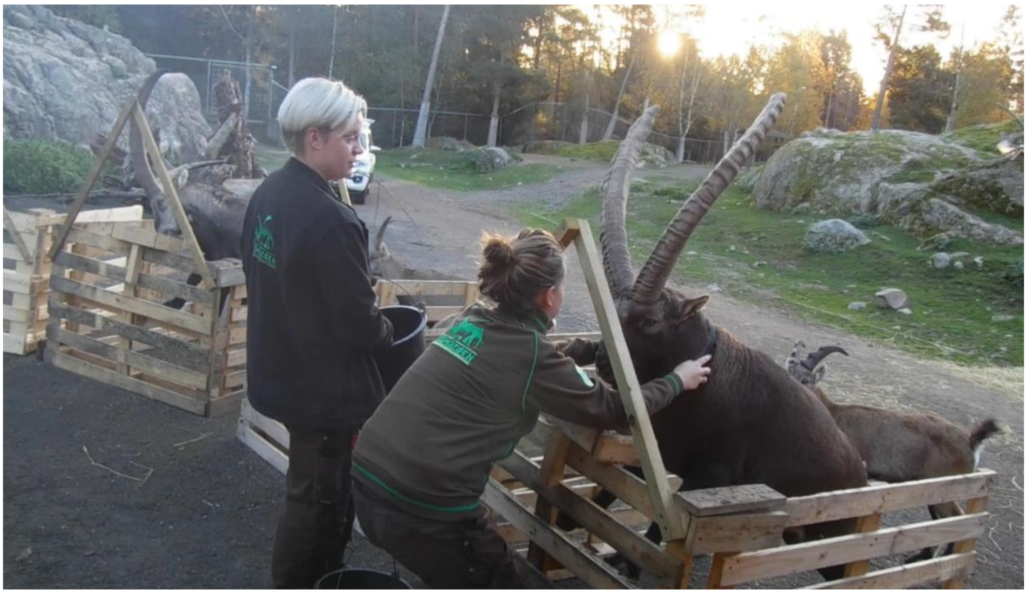

Figure S4: Holding the collar around a male ibex neck while he stands in the protective station, one trainer holds the collar while the second provides the reward (step 7: Photo; Pieter Giljam)

Table S3: Total time observed of each behaviour for each individual pygmy goat (G) or Alpine ibex (IB) in seconds.

	Individual ID														
**Behaviour**	**IB01**	**IB02**	**IB03**	**IB04**	**IB05**	**IB06**	**G01**	**G02**	**G03**	**G04**	**G05**	**G06**	**G07**	**G08**	**G09**
Aggression	25.37	16.14	5.79	0.9	5.95	4.88	6.43	3.45	0	5.02	1.79	3.38	3.3	4.44	1.57
Browsing	0	0	0	0	0	0	128.57	28.66	20.09	7	1.97	4.71	3	0.6	0.77
Climbing	8.21	3.22	6.25	10.17	14.03	2.16	0	0	0	0	0	0	0	0	0
Eating	108.99	59.05	159.19	121.37	248.33	113.49	80.21	134.87	91.4	261.44	84.67	239.02	223.83	148.61	64.28
Grooming	11.46	7.02	0.63	1.79	0.64	2.71	1.87	1.1	5.36	1.12	1.46	19.43	8.01	4.54	0
Resting	209.32	150.24	61.01	93.87	39.25	62.9	0	0	59.02	51.92	279.86	216.69	57.73	121.14	0
Running	2.37	0.61	8.08	3.89	11.87	6.43	3.12	5.7	0.52	4.61	3.09	2.9	2.84	2.18	0.53
Shaking	1.45	5.23	2.53	3.38	0.55	3.27	1.38	1.15	0	0.63	0.76	0	0.89	0.92	0
Standing	122.53	301.38	88.31	132.47	98.47	125.95	155.33	102.45	55.29	74	98.7	92.6	93.73	129.4	63.94
Trotting	3.04	0.21	12.66	6.39	5.43	4.99	14.73	9.79	0	3.62	7.6	4.39	3.68	7.36	1.81
Walking	95.99	93.79	99.27	120.41	106.82	86.45	86.2	81.52	29.68	82.65	58.76	90.28	62.01	67.37	37.08
Total	588.73	636.89	443.72	494.64	531.34	413.23	477.84	368.69	261.36	492.01	538.66	673.4	459.02	486.56	169.98
