# Supplementary material for: Limitations of using surrogates for behaviour classification of accelerometer data: refining methods using random forest models in Caprids
Source: Mov Ecol. 2021 Jun 7;9:28. doi: 10.1186/s40462-021-00265-7 (PMC8186069; doi:10.1186/s40462-021-00265-7)
Supplement: Supplementary file 2 — Additional file 2 Methods for building and refining random forest models to predict the behaviour of Alpine ibex and pygmy goats. Table S4. A list of the accelerometry and magnetometry variables that are used or calculated for the random forest model. Including the name, and label, the description of the variable and its calculation. Figure S4. Recursive feature elimination plots showing the cross-validated model accuracy when a different number of acceleration and magnetometry variables are included in the random forest models for classifying the behaviours of (a) Alpine ibex and (b) pygmy goat. Figure S5. Random forest error plots across 500 trees for classifying each of the nine behavioural states (Aggression, Browsing (pygmy goats only), Climbing (Alpine ibex only), Grazing, Grooming, Lying down, Running, Shaking, Standing, Trotting and Walking) and Out-of-bag (OOB) error estimates for each different model at 10 Hz for both species (a,b) including the models with: (c,d) balanced observations and (e,f) reduced behaviour classes. Figure S6. Random forest error plots across 500 trees for classifying each of the nine behavioural states including terrain slope for locomotion behaviours (Aggression, Browsing (pygmy goats only), Climbing (Alpine ibex only), Grazing, Grooming, Lying down, Running, Shaking, Standing, Trotting and Walking) and Out-of-bag (OOB) error estimates, for (A) Alpine ibex and (B) pygmy goats. Table S5. The variable reduction process to reach the final selected model. [file 40462_2021_265_MOESM2_ESM.pdf]

Additional file 2: Methods for building and refining random forest models to predict the behaviour of Alpine ibex and pygmy goats.

Table S4: A list of the accelerometry and magnetometry variables that are used or calculated for the random forest model. Including the name, and label, the description of the variable and its calculation.

Variable	Description	Reference
**Static acceleration (staX, stay, staZ)**	The static acceleration of each axis, representing the orientation of the device and thus the subjects' posture. Calculated as the running mean of acceleration for each orthogonal axis over 2 seconds.	
**Pitch and Roll**	To define the posture of the plane and upright position the 3D orientation towards gravity was converted to angles, of the X axis representing 'sway' movement (Pitch) and Y axis representing 'surge' movement (Roll). Calculated as the arcsine of the X and Y axis respectively.	(Wilson, Shepard and Liebsch, 2008)
**Dynamic acceleration (dynX, dynY, dynZ)**	The dynamic acceleration of each axis representing the body movement of the animal. Calculated as the static acceleration subtracted from the raw acceleration for each axis.	
**Overall Dynamic Body Acceleration (ODBA)**	A measure of the total body acceleration. The sum of the absolute dynamic acceleration of all three orthogonal axes.	(Halsey, Shepard and Wilson, 2011)
**Vectorial Dynamic Body Acceleration (VeDBA)**	A second measure of total body acceleration which provides values closer to the true acceleration experienced. Unlike ODBA it is not sensitive to device orientation. It is calculated as the square root of the sum of each acceleration axes squared.	(Bidder, Qasem and Wilson, 2012; Qasem *et al.*, 2012)
**VeDBA smoothed (smVeDBA)**	To remove noise and reduce the variation in the VeDBA signal, an additional variable of smoothed VeDBA was used. VeDBA was smoothed using a running mean of 1 sec, was calculated over 1 second using a running mean to remove the variation in VeDBA at 40Hz.	
**Partial dynamic body acceleration (PDBA X, PDBA Y, PDBA Z)**	The absolute values of acceleration, providing the amplitude of acceleration for each axis. Calculated by returning the absolute positive value of acceleration.	(Fehlmann, O'Riain, Hopkins, *et al.*, 2017)
**VeDBA:PDBA ratio (ratioX, ratioY, ratioZ)**	The ratio of VeDBA to PDBA for each axis which gives the contribution of each axis acceleration to VeDBA. Calculated by dividing VeDBA by PDBA.	
**Differential acceleration (difX, difY, difZ)**	The rate of change of acceleration over time for each axis. This was calculated for each axis, as the change in acceleration over 5 data points (0.125 sec).	(Ydesen *et al.*, 2014; Shuert, Pomeroy and Twiss, 2018)
**Jerk**	The differential of acceleration for the three axes, which is the overall change in acceleration, is referred to as Jerk. Calculated by taking the square root of the sum of all three axes differential multiplied by the sampling rate.	
**First power spectrum density and maximum frequency (PSD1X, PSD1Y, PSD1Z and freq1X, freq1Y, freq1Z)**	The amplitude and frequency of oscillations was calculated using a Fast Fourier Transformation (FFT) analysis. The first power spectrum density (PSD) and maximum frequency for each axis over a period of two seconds [22-33], was calculated using code adapted from Fehlmann *et al*. 2017.	(Fehlmann, O'Riain, Hopkins, *et al.*, 2017)
**Second power spectrum density and maximum frequency (PSD2X, PSD2Y, PSD2Z and freq2X, freq2Y, freq2Z)**	As described above.	
**Magnetic smoothed (magX, magY,magZ)**	The magnetic orientation of the device in relation to the magnetic field of the earth for three axes, smoothed over 40 data points (1 sec).	(Williams *et al.*, 2017; Chakravarty *et al.*, 2019)
**Magnetic vectoral sum smoothed (MagVecsum)**	The smoothed sum of the vectoral magnetometry for three axes, depicting overall absolute change in magnetic orientation.	
**Magnetic pitch and roll (magpitch, magroll)**	A measure of magnetic posture measured as angles using the plane and upright position from the X axis (pitch) and Y axis (roll), calculated using the arcsine of each axis respectively.	

(a)

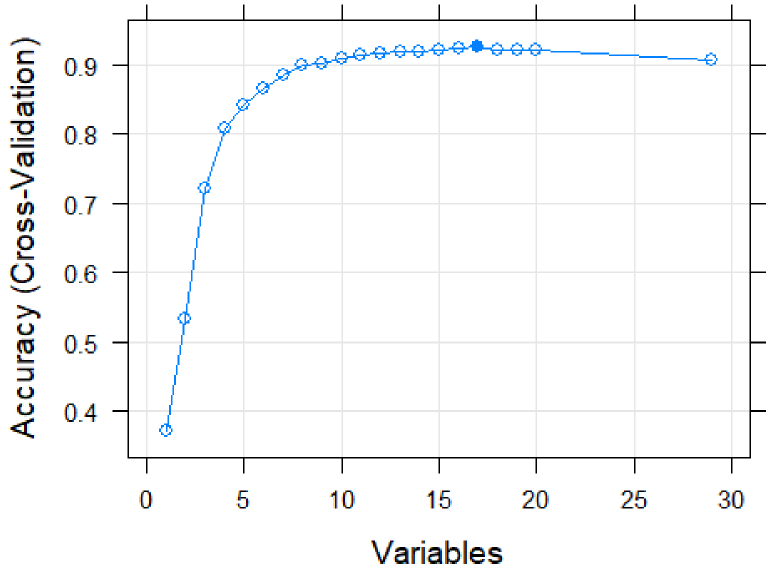

(b)

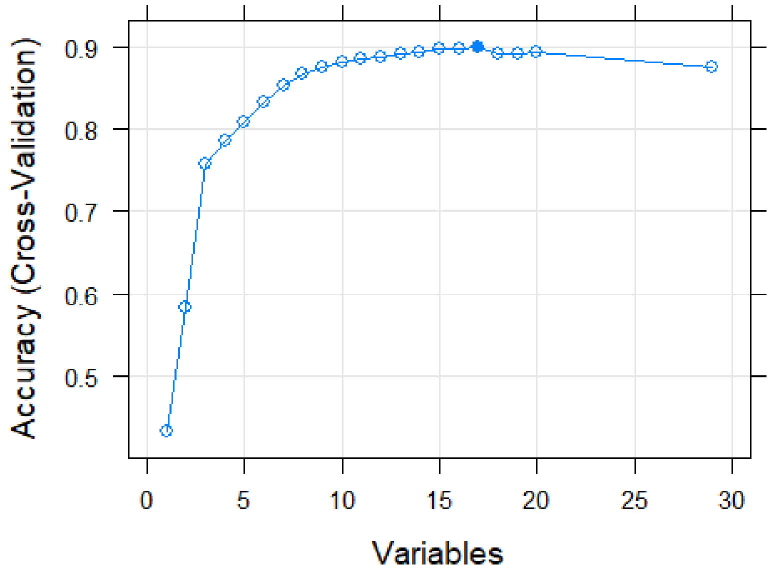

Figure S4: Recursive feature elimination plots showing the cross-validated model accuracy when a different number of acceleration and magnetometry variables are included in the random forest models for classifying the behaviours of (a) Alpine ibex and (b) pygmy goat.

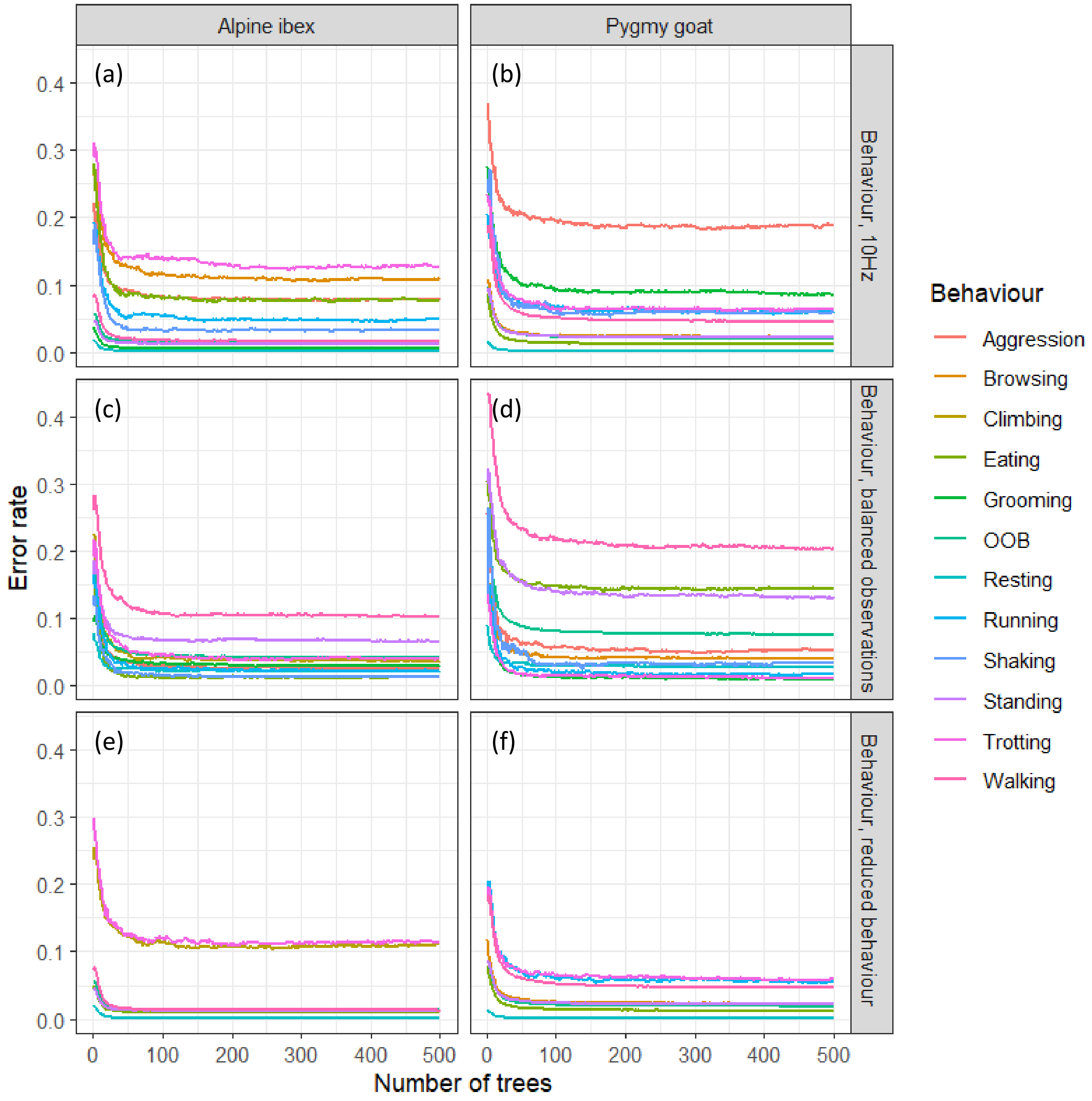

Figure S5: Random forest error plots across 500 trees for classifying each of the nine behavioural states (Aggression, Browsing (pygmy goats only), Climbing (Alpine ibex only), Grazing, Grooming, Lying down, Running, Shaking, Standing, Trotting and Walking) and Out-of-bag (OOB) error estimates for each different model at 10Hz for both species (A,B) including the models with: (C,D) balanced observations and (E,F) reduced behaviour classes.

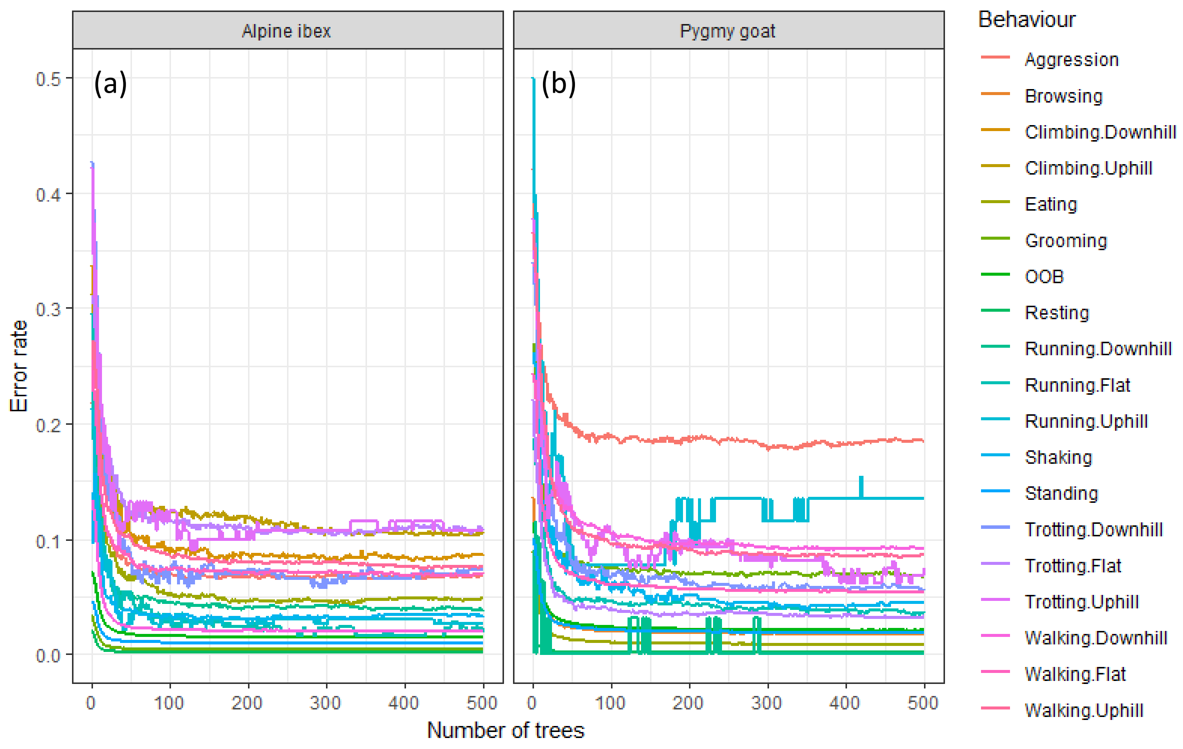

Figure S6: Random forest error plots across 500 trees for classifying each of the nine behavioural states including terrain slope for locomotion behaviours (Aggression, Browsing (pygmy goats only), Climbing (Alpine ibex only), Grazing, Grooming, Lying down, Running, Shaking, Standing, Trotting and Walking) and Out-of-bag (OOB) error estimates, for (A) Alpine ibex and (B) pygmy goats.
