# Supplementary material for: Limitations of using surrogates for behaviour classification of accelerometer data: refining methods using random forest models in Caprids
Source: Mov Ecol. 2021 Jun 7;9:28. doi: 10.1186/s40462-021-00265-7 (PMC8186069; doi:10.1186/s40462-021-00265-7)
Supplement: Supplementary file 4 — Additional file 4: Figure S6. The importance of each variable ordered by mean Gini decrease for the model predicting behaviours including slope of terrain; (a Pygmy goats with ‘Pitch’ as the most important variable and (b) Alpine ibex with ‘Static X’ as the most important variable. Table S5. The median and 1st and 3rd quantile of acceleration, for each behaviour and species, for the three variables that are in the top 5 most important variables for predicting behaviour of both pygmy goats and Alpine ibex. Table S6. Confusion matrix showing the observed behaviours and predicted behaviours when using a random forest model built using pygmy goat training dataset and tested on the Alpine ibex training data set. Italicised cells are the true positives where the behaviour has been correctly predicted. Table S7. Confusion matrix showing the observed behaviours and predicted behaviours, including the gradient of terrain for locomotion behaviours, when using a random forest model built using pygmy goat training dataset and tested on the Alpine ibex training data set. Italicised cells are the true positives where the behaviour has been correctly predicted. (Downhill = D, Flat = F, Uphill = U). [file 40462_2021_265_MOESM4_ESM.pdf]

Additional file 3: Random forest model results

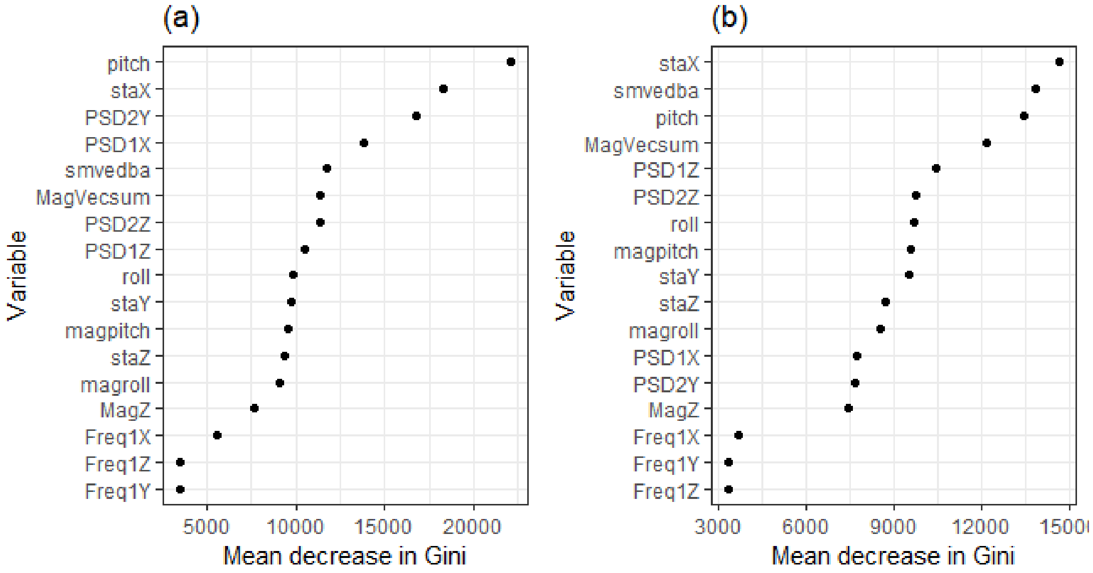

Figure S6: The importance of each variable ordered by mean Gini decrease for the model predicting behaviours including slope of terrain; (a Pygmy goats with 'Pitch' as the most important variable and (b) Alpine ibex with 'Static X' as the most important variable.

Table S5: The median and 1st and 3rd quantile of acceleration, for each behaviour and species, for the three variables that are in the top 5 most important variables for predicting behaviour of both pygmy goats and Alpine ibex.

		Pitch			Static X			Smoothed VeDBA		
**Species**	**Behaviour**	**Median**	**1st quantile**	**3rd quantile**	**Median**	**1st quantile**	**3rd quantile**	**Median**	**1st quantile**	**3rd quantile**
Alpine ibex	Aggression	5.89	-10.93	18.28	-0.10	-0.31	0.18	0.11	0.06	0.29
	Eating	11.09	5.11	15.43	-0.19	-0.26	-0.08	0.07	0.05	0.10
	Resting	-24.38	-30.11	-16.35	0.39	0.27	0.49	0.03	0.02	0.03
	Running	-23.41	-32.65	-15.68	0.40	0.28	0.53	0.99	0.67	1.34
	Grooming	-14.21	-28.43	-1.76	0.24	0.03	0.45	0.10	0.07	0.15
	Shaking	-11.90	-19.85	-3.01	0.21	0.05	0.35	0.81	0.29	1.72
	Standing	-28.55	-35.37	-19.25	0.45	0.32	0.55	0.03	0.03	0.04
	Trotting	-21.12	-26.87	-13.98	0.35	0.23	0.43	0.38	0.27	0.58
	Walking	-18.79	-25.81	-8.92	0.31	0.15	0.41	0.16	0.12	0.23
	Climbing	-8.55	-22.33	1.72	0.15	-0.03	0.36	0.30	0.18	0.56
Pygmy goat	Aggression	16.40	-0.25	32.31	0.26	0.00	0.48	0.36	0.22	0.59
	Browsing	39.46	22.76	62.99	0.60	0.34	0.84	0.08	0.05	0.14
	Eating	-3.95	-10.95	7.92	-0.07	-0.18	0.13	0.09	0.07	0.14
	Resting	36.27	27.50	40.51	0.56	0.44	0.60	0.03	0.02	0.03
	Running	19.27	11.94	27.64	0.33	0.20	0.47	0.55	0.32	0.94
	Grooming	21.28	11.01	30.05	0.34	0.18	0.48	0.12	0.09	0.17
	Shaking	17.97	11.60	27.88	0.29	0.19	0.46	0.86	0.43	1.44
	Standing	30.61	21.06	39.22	0.48	0.33	0.60	0.04	0.03	0.06
	Trotting	13.56	8.69	18.61	0.23	0.15	0.30	0.30	0.23	0.39
	Walking	15.00	6.75	24.00	0.25	0.11	0.39	0.17	0.13	0.23

Table S6: Confusion matrix showing the observed behaviours and predicted behaviours when using a random forest model built using pygmy goat training dataset and tested on the Alpine ibex training data set. Italicised cells are the true positives where the behaviour has been correctly predicted.

	**Observed behaviour**					
**Predicted behaviour**	**Grazing**	**Resting**	**Running**	**Standing**	**Trotting**	**Walking**
Grazing	*484.4*	10.5	0	120.3	1.4	206.2
Resting	0	*12.2*	0	4.1	0	0
Running	0	0	*296.7*	0.6	131.4	16
Standing	55.2	567.5	2	*451.2*	0.2	3.2
Trotting	0.1	0	16.6	0.1	*37.1*	5.7
Walking	50.6	0.1	17.2	14	157.1	*359.2*

Table S7: Confusion matrix showing the observed behaviours and predicted behaviours, including the gradient of terrain for locomotion behaviours, when using a random forest model built using pygmy goat training dataset and tested on the Alpine ibex training data set. Italicised cells are the true positives where the behaviour has been correctly predicted. (Downhill = D, Flat = F, Uphill = U)

	**Observed behaviour**											
**Predicted behaviour**	**Eating**	**Resting**	**Running (D)**	**Running (F)**	**Running (U)**	**Standing**	**Trotting (D)**	**Trotting (F)**	**Trotting (U)**	**Walking (F)**	**Walking (D)**	**Walking (U)**
**Eating**	*2686.3*	29.1	0	0.5	0	197.7	1.8	6.7	0	496	93.6	64.5
**Resting**	0	*486.5*	0	0	0	606.3	0	0	0	0.1	0	0
**Running (D)**	0	0	*0*	0	0	0	0	0	0	0	0	0
**Running (F)**	0.5	0	20.8	*115.3*	14.6	2.3	6.4	36.3	3.4	17.1	2.6	0.9
**Running (U)**	0	0	0	0	*0*	0	0	0	0	0	0	0
**Standing**	87.5	3192.6	0.8	13.9	3.4	*4367.5*	0.7	3.5	0.2	449.5	37.8	62
**Trotting (D)**	0	0	0	0	0	0	*0*	0	0	0	0	0
**Trotting (F)**	0.1	0	1	6.1	0	0.3	1.5	*17.8*	2	47.8	15.9	6.1
**Trotting (U)**	0	0	0	0.5	0	0	0	0.9	*0*	0.1	0	0
**Walking (F)**	18.7	1.2	1.1	19.4	2.8	58.7	13.9	85.3	7.1	*1712.5*	210	241.7
**Walking (D)**	2.8	0	0	1.6	0	2.3	1.6	3.4	0	31.7	*16.8*	6.5
**Walking (U)**	0	0	0	0	0	0.1	0	2.8	0	61.2	17.3	*15.3*
